# Treatment Outcomes With Novel Targeted and Immunotherapeutic Regimens in CAYA Hodgkin Lymphoma: A Retrospective Study

**DOI:** 10.1002/cai2.70050

**Published:** 2026-03-16

**Authors:** Mengqing Xie, Meng Yuan, Mengwei Ren, Jing Tian, Shengyu Zhou, Xiaohui He, Yan Qin, Peng Liu, Jianliang Yang, Mengyuan Han, Fei Ma, Sheng Yang, Sidan Li

**Affiliations:** ^1^ Department of Medical Oncology National Cancer Center/National Clinical Research Center for Cancer/Cancer Hospital, Chinese Academy of Medical Sciences and Peking Union Medical College Beijing China; ^2^ Hematology Center, Beijing Children's Hospital Capital Medical University Beijing China; ^3^ Department of Pediatric, Beijing Jishuitan Hospital Capital Medical University Beijing China; ^4^ Department of Pediatric, Beijing Shijitan Hospital Capital Medical University Beijing China

**Keywords:** adolescent and young adult, brentuximab vedotin, Hodgkin lymphoma, immune checkpoint inhibitors

## Abstract

**Background:**

To evaluate the efficacy and safety of novel chemotherapy regimens, including brentuximab vedotin (BV) and immune checkpoint inhibitors (ICIs), in children, adolescents, and young adults (CAYA) with Hodgkin lymphoma (HL).

**Methods:**

A retrospective analysis was conducted on untreated Stage IIB (bulky), III, or IV HL patients (≤ 24 years) who were admitted to Cancer Hospital of the Chinese Academy of Medical Sciences and Beijing Children's Hospital, Capital Medical University, from 2017 to 2024. The cohort compared conventional regimens with BV‑based therapies (BV‑AVD ± R). Furthermore, the efficacy and safety of ICIs were evaluated in relapsed/refractory cHL patients receiving second‑line or later therapy.

**Results:**

In this comparison between BV and conventional regimens, 182 patients were enrolled. The median follow‐up was 38 months. No significant differences were observed in objective response rate (ORR) (96.4% *vs* 95.4%, *p* = 0.927) or progression‐free survival (PFS) rate (92.8% *vs* 95.3%, *p* = 0.466).However, the incidence of Grade 4 hematologic toxicity was significantly higher with conventional therapy compared to BV‐AVD±R (61.2% *vs* 25.6%, *p* < 0.001). The BV‐AVD group alone achieved a significantly higher ORR than the BV ‐AVD+R group (100% vs. 93.9%, *p* = 0.047), while there was no significant difference in PFS rate. However, the BV‐AVD + R group exhibited a significantly higher incidence of Grade 3–4 hematologic toxicities (78.8% vs. 20.0%, *p* = 0.023). Immune checkpoint inhibitors was well‐tolerated with infrequent adverse events, and 18/19 cases (94.7%) demonstrated treatment efficacy.

**Conclusion:**

For patients with stage II Hodgkin lymphoma presenting with bulky disease, as well as those with stage III–IV disease, the BV‐AVD regimen is recommended as first‐line therapy, given its more favorable toxicity profile.Although ICIs represent a promising therapeutic approach, existing studies are constrained by limited sample sizes. Future research should focus on expanding patient cohorts to refine treatment strategies and ultimately improve clinical outcomes.

## Graphical abstract

Compared to conventional chemotherapy, treatment with BV‐AVD ± R in CAYA Hodgkin lymphoma patients resulted in comparable response rates (ORR: 96.4% vs. 95.4%) but with a marked reduction in Grade 4 hematologic toxicity (25.6% vs. 61.2%). BV‐AVD monotherapy yielded a higher ORR than BV‐AVD + R (100% vs. 93.9%). Adult prognostic scores better predicted outcomes in the 6–24‐year cohort (AUC: 0.696 vs. 0.573). In R/R patients, ICIs achieved a 94.7% ORR with a favorable safety profile.

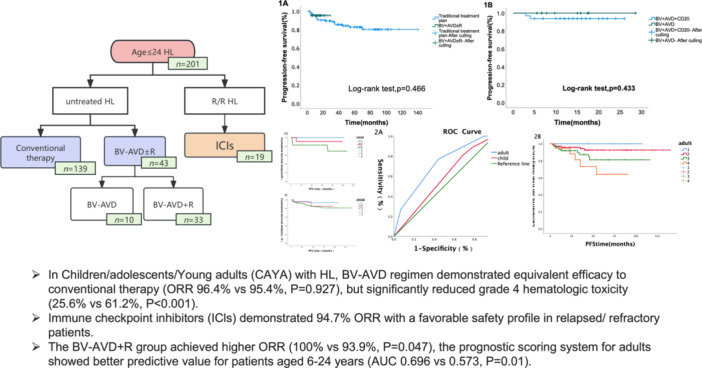

**Abbreviations**
AUC: area under the curve
AYA: adolescent and young adult
BV: brentuximab vedotin
CAYA: children, adolescents, and young adults
COG: Children's Oncology Group
CR: complete response
HL: Hodgkin lymphoma
ICIs: immune checkpoint inhibitors
MMAE: monomethyl auristatin E
ORR: overall response rate
PFS: progression‐free survival
PR: partial response
ROC: receiver operating characteristic
R/R: relapsed/refractory
SAEs: serious adverse events

## 1 Background

The epidemiological profile of Hodgkin lymphoma (HL), a common lymphoma subtype, has attracted considerable global attention. Globocan 2022 data report a worldwide age‐standardized HL incidence rate of approximately 0.98 per 100,000 in 2020 [1], with substantial regional variations. Incidence is relatively higher in high‐income countries, reaching 4–6 per 100,000 in some developed European nations and the United States. Conversely, rates are lower in Asia; China reports an age‐standardized incidence of approximately 0.8–1.2 per 100,000. With population aging and changing environmental factors, China's HL incidence has demonstrated a steady annual increase, raising significant public health concern. HL incidence exhibits a distinct bimodal age distribution, predominantly affecting individuals under 30 years and those over 60 years [2, 3].

Conventional chemotherapy regimens remain the cornerstone of first‐line management for newly diagnosed children, adolescents, and young adults (CAYA) HL patients. Commonly employed protocols include risk‐adapted pediatric regimens (such as ABVE‐PC, COPP/ABV, and Ara‐C/VP‐16‐based regimens) and the ABVD regimen, more frequently used in adults [4, 5]. However, therapeutic efficacy may be inadequate for some patients, and intensive pediatric therapies carry significant toxicity risks. Advances in medical research are facilitating the integration of novel strategies incorporating brentuximab vedotin (BV) and immune checkpoint inhibitors (ICIs) into clinical practice [6]. Studies suggest regimens containing these novel agents offer potential advantages in enhancing therapeutic efficacy and improving prognosis. For example, incorporating BV into first‐line treatment for high‐risk pediatric HL demonstrates favorable tolerability [5, 7], significantly reducing radiotherapy dependence while improving outcomes. Although ICIs demonstrate promising efficacy in adult HL and preliminary studies explore their use in pediatric HL, real‐world data on ICI application specifically within the pediatric population remain scarce [6].

The objectives were to (1) evaluate the efficacy and safety of novel BV‐containing chemotherapy regimens versus conventional chemotherapy in newly diagnosed CAYA HL, (2) assess whether adding an anti‐CD20 monoclonal antibody to BV‐AVD chemotherapy provides additional clinical benefit while maintaining acceptable safety, and (3) summarize treatment response and safety profiles of ICIs in relapsed/refractory (R/R) HL patients receiving at least two prior lines of therapy. (4) This study aims to assess the predictive value of pediatric and adult risk assessment systems for CAYA with Hodgkin's lymphoma.

## 2 Methods

### 2.1 Case Data

This retrospective study analyzed 182 previously untreated cHL cases aged ≤ 24 years and 19 cases with R/R disease. Patients were treated at the Cancer Hospital of the Chinese Academy of Medical Sciences and Beijing Children's Hospital, Capital Medical University, between January 1, 2017, and December 1, 2024. The cohort comprised 73 previously untreated HL patients and 9 R/R HL patients from the Cancer Hospital of the Chinese Academy of Medical Sciences, alongside 109 previously untreated patients and 10 R/R patients from Beijing Children's Hospital, Capital Medical University [7]. Staging was performed according to the 2014 Lugano classification criteria for comprehensive assessment of tumor size, extent, invasion sites, and distant metastasis [8]. PET‐CT utilization has become increasingly prevalent in staging, with studies demonstrating its ability to more accurately detect small metastases and provide enhanced staging information through quantitative analysis of metabolic parameters such as standardized uptake value (SUV) [9].

The pediatric prognostic scoring system categorized patients into low‐risk, intermediate‐risk, and high‐risk groups based on disease stage, presence of bulky disease, and B symptoms, consistent with the 2019 Chinese Guidelines for Diagnosis and Treatment of Pediatric HL [4]. For adult HL patients, prognostic assessment employed the adult prognostic scoring system. Early‐stage (I–II) patients were stratified using EORTC/GHSG/NCCN criteria. Advanced‐stage (III–IV) patients were risk‐stratified using the International Prognostic Score (IPS).

Inclusion criteria: Age ≤ 24 years; histopathologically confirmed HL (for R/R patients, a second histopathological confirmation of HL was required); specific disease stage (untreated patients: Stage IIB with bulky disease, Stage III, or Stage IV; R/R patients: no prior treatment with ICIs); voluntarily provided written informed consent.

Exclusion criteria: Prior history of hematopoietic stem cell transplantation; missing key clinical data.

### 2.2 Comparison of Conventional Chemotherapy Regimens and BV‐Containing Regimens

Patients with untreated Stage IIB HL exhibiting bulky disease or Stage III/IV HL were included. Conventional chemotherapy encompassed guideline‐recommended adult and pediatric regimens. Adult regimens included ABVD (doxorubicin 25 mg/m^2^, bleomycin 10 mg/m^2^, vincristine 3 mg/m^2^ [capped at 5 mg], dacarbazine 375 mg/m^2^ administered on Days 1 and 15 of 28‐day cycles for 4–6 cycles, with or without radiotherapy) and BEACOPP. PET2‐positive patients after two ABVD cycles received BEACOPP escalation, followed by 2–4 consolidation cycles ± radiotherapy. Pediatric regimens followed the 2019 Standardized Diagnosis and Treatment Protocol using risk‐adapted strategies: Low‐risk patients (Stage IA/IIA non‐bulky) received ABVE/PC‐COPP/ABV or ABVEPC‐COPP/ABV for four cycles. Intermediate‐risk patients (IB/IIIA non‐bulky) received CycleA‐COPP/ABV‐CycleC‐Cyclea‐COPP/ABV‐CycleC for six cycles plus radiotherapy. High‐risk patients received CycleA‐COPP/ABV‐CycleC‐CycleA‐COPP/ABV‐CycleC, with CycleC initiation permitted for symptomatic Group B. The BV + AVD ± R regimen comprised BV 1.2 mg/kg (max 120 mg) Days 1 and 15; doxorubicin 25 mg/m^2^ Days 1 and 15; vindesine 3 mg/m^2^ (max 5 mg) Days 1 and 15; dacarbazine 375 mg/m^2^ Days 1 and 15; and rituximab 375 mg/m^2^ Days 2 and 16 for six cycles, with low‐dose involved‐site radiotherapy considered for incomplete metabolic response.

### 2.3 BV‐AVD + Rituximab Versus BV‐AVD

This comparison included patients with untreated Stage IIB HL with bulky disease or Stage III/IV HL. The evaluated regimens (BV + AVD ± R) were identical to those detailed in the preceding section.

### 2.4 ICIs‐Containing Regimens

Patients with R/R HL receiving ICIs‐containing regimens were analyzed. ICIs were administered according to prescribing information or clinical trial protocols: for example, pembrolizumab 2 mg/kg every 3 weeks and camrelizumab 3 mg/kg every 3 weeks. All doses remained within approved maximum limits for adults.

### 2.5 Evaluation of Prognostic Models and Confounder Adjustment

To assess the discriminative ability of the IPS and COG scoring systems, we constructed time‐dependent receiver operating characteristic (ROC) curves at 5 years. The area under the curve (AUC) was calculated for each model, with comparisons made via DeLong's test. Subsequently, Kaplan–Meier survival curves were generated for risk strata defined by each system and compared using the log‐rank test. Finally, multivariable Cox regression was performed to adjust for potential confounders, including age, treatment regimen, and radiotherapy.

### 2.6 Efficacy Evaluation

Treatment response was assessed according to the 2014 Lugano Classification criteria for lymphoma [9, 10].

Objective response rate (ORR): The proportion of patients achieving either a complete response (CR) or a partial response (PR), calculated as (number of CR + number of PR)/total number of evaluable patients × 100%.

CR:PET‐CT criteria: A Deauville 5‐point scale score of 1, 2, or 3 for involved nodal and extranodal sites, with or without a residual mass, and no evidence of FDG‐avid disease.

CT criteria: Target lymph nodes must have a long‐axis diameter ≤ 1.5 cm; disappearance of all extranodal lesions; resolution of non‐measurable disease; normalization of organ enlargement; and normal bone marrow morphology.

PR:PET‐CT criteria: A Deauville score of 4 or 5 with a clinically significant reduction in FDG uptake compared to the baseline scan, in the absece of new lesions. This means no new FDG‐avid lesions have emerged during or after treatment, with causes other than lymohoma (such as infection or inflammation) having been ruled out for the observed uptake.

CT criteria: A ≥ 50% decrease from baseline in the sum of the products of the perpendicular diameters (SPD) of up to six target lesions (nodal and extranodal).

### 2.7 Adverse Events Monitoring

Patients underwent systematic surveillance for infusion reactions, gastrointestinal symptoms, and sensory abnormalities post‐treatment, with particular attention to neurotoxicity, pulmonary toxicity, and hematotoxicity. Adverse events were graded according to CTCAE v5.0 [11]. Long‐term quality‐of‐life impacts were documented, including persistent reproductive and cardiovascular sequelae.

### 2.8 Follow‐Up

Patients were followed through December 1, 2024 (final cutoff), with assessments encompassing primary disease status and treatment‐related complications, including growth/development and cardiopulmonary function. Follow‐ups occurred quarterly during the first two post‐treatment years, with subsequent intervals adjusted clinically. Telemedicine implementation enhanced follow‐up efficiency and compliance. The primary endpoints of this study were defined as follows:

OS was calculated from the initiation of first‐line treatment to death from any cause, with living patients censored at their last follow‐up.

Progression‐free survival (PFS) was defined as the time from the start of first‐line treatment (either conventional chemotherapy or BV‐containing regimens) to the first documented disease progression, relapse, or death from any cause, with event‐free patients censored at their last follow‐up date.

### 2.9 Statistical Analysis

SPSS 22.0 facilitated all analyses. Categorical variables were compared using chi‐square tests. Non‐parametric tests analyzed non‐normally distributed continuous variables. Kaplan–Meier methodology estimated overall survival with log‐rank tests comparing survival curves. Cox proportional hazards models adjusted for age, stage, and treatment regimen imbalances. Binary outcomes underwent logistic regression with covariate adjustment. Significance was defined as *p* < 0.05. The prognostic performance of the two models was compared using ROC curve analysis, and the accuracy of the models was evaluated based on the AUC.

## 3 Results

### 3.1 Comparison Between Conventional Chemotherapy and BV‐Containing Regimens

#### 3.1.1 Baseline Characteristics

A cohort of 182 previously untreated HL patients with Stage IIB bulky disease, Stage III, or Stage IV disease was analyzed. Of these, 139 received conventional first‐line therapy (according to aforementioned pediatric/adult cHL protocols), while 43 underwent BV‐AVD ± R chemotherapy. Baseline characteristics are detailed in Table 1. The groups demonstrated comparable distributions regarding age, sex, histologic subtype, disease stage, bulky disease status, radiotherapy utilization, and risk stratification (all *p* > 0.05).

**Table 1:** Comparison of baseline characteristics between the conventional therapy group and BV‐AVD ± R group.

Characteristics		Conventional therapy (*n* = 139)	BV‐AVD ± R (*n* = 43)	*p*
Age (median, min–max)		14 (3–24)	12 (5–24)	0.302
Sex (%)	Male	91 (65.5)	27 (62.8)	0.748
	Female	48 (34.5)	16 (37.2)	
Histology (%)	Nodular sclerosis	66 (47.5)	18 (41.9)	0.162
	Mixed cellularity	56 (40.3)	14 (31.6)	
	Lymphocyte‐rich	5 (3.6)	2 (4.7)	
	Lymphocyte‐depleted	0 (0)	0 (0)	
	Unspecified	12 (8.6)	9 (20.9)	
Stage (%)	I	0 (0)	0 (0%)	0.308
	II	50 (36.0)	12 (27.9)	
	III	41 (29.5)	18 (41.9)	
	IV	48 (34.5)	13 (30.2)	
B symptoms (%)	Yes	68 (48.9)	14 (32.6)	0.059
	No	71 (51.1)	29 (67.4)	
Bulky disease (%)	Yes	85 (61.2)	20 (46.5)	0.089
	No	54 (38.8)	23 (53.5)	
Radiotherapy (%)	Yes	25 (18.0)	11 (25.6)	0.275
	No	114 (82.0)	32 (74.4)	
Pediatric risk group (%)	Low risk	0 (0.0)	0 (0.0)	0.053
	Intermediate risk	12 (8.6)	9 (20.9)	
	High risk	127 (91.4)	34 (79.1)	
Adult risk group (%)	Favorable prognosis	0 (0.0)	0 (0.0)	0.436
	Unfavorable prognosis	48 (34.5)	12 (27.9)	
	IPS ≤ 3	72 (51.8)	27 (62.8)	
	IPS ≥ 4	19 (13.7)	4 (9.3)	

#### 3.1.2 Response Rates and Survival Analysis

**Figure 1:**
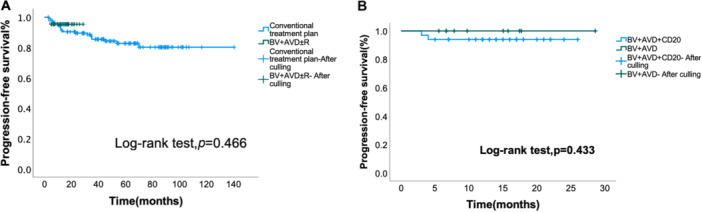
Progression‐free survival (PFS) curves. (A) Comparison of PFS curves between conventional therapy and BV‐AVD ± R. (B) Comparison of PFS curves between BV‐AVD + R and BV‐AVD.

The conventional treatment group achieved an overall response rate (ORR) of 96.4% (CRR 82.0%), while the BV + AVD ± R group showed an ORR of 95.4% (CRR 86.0%), with no significant ORR difference (*p* = 0.927). At a median overall follow‐up of 38 months (range: 3–140 months), the median follow‐up was 50 months (range: 3–140 months) for the conventional chemotherapy group and 14 months (range: 3–29 months) for the BV + AVD ± R group. Two‐year PFS was 92.8% versus 95.3%, respectively. Kaplan–Meier analysis revealed no significant PFS difference (Figure 1A).

#### 3.1.3 Safety

Among 182 patients, 175 adverse events occurred. Grade 3–4 hematological toxicity incidence was comparable overall, but Grade 4 events were significantly higher with conventional treatment (61.2% vs. 25.6%, *p* < 0.001). Cardiac impairment incidence was higher with conventional regimens (26.6% vs. 2.3%, *p* < 0.01), though no Grade > 3 events occurred. Conventional treatment showed higher infection rates without a significant difference in Grade ≥ 3 events. No BV‐associated peripheral neurotoxicity was observed (Table 2).

**Table 2:** Comparison of adverse events between first‐line conventional chemotherapy and the BV‐AVD ± R regimen.

	Conventional therapy (*n* = 139)		BV‐AVD ± R (*n* = 43)		*p*	
	Incidence	Grade 3–4 incidence	Incidence	Grade 3–4 incidence	Incidence	Grade 3–4 incidence
Adverse events (%)	132 (95.0)	101 (72.7)	43 (100)	32 (74.4)	0.133	0.820
Hematological toxicity (%)	120 (86.3)	98 (70.5)	39 (90.7)	28 (65.1)	0.451	0.504
Liver impairment (%)	48 (34.5)	11 (7.9)	18 (42.9)	6 (14.0)	0.326	0.234
Cardiac impairment (%)	37 (26.6)	0 (0)	1 (2.3)	0 (0)	< 0.01	—
GI symptoms (%)	26 (18.7)	2 (1.4)	5 (11.6)	0 (0)	0.281	0.521
Infection (%)	47 (33.8)	15 (10.8)	6 (14.0)	3 (7.0)	0.012	0.571
Neurotoxicity (%)	3 (2.2)	1 (0.7)	2 (4.7)	0 (0)	0.082	0.361
Mucositis (%)	10 (7.2)	0 (0)	1 (2.3)	0 (0)	0.338	—

### 3.2 Comparison Between BV‐AVD + R and BV‐AVD

#### 3.2.1 Baseline Characteristics

The analysis included 43 patients: 33 received BV‐AVD + anti‐CD20 antibody (R), and 10 received BV‐AVD alone. The BV‐AVD + R group was significantly younger (median 11 vs. 19.5 years, *p* = 0.003). Groups were comparable in sex, histology, stage, B symptoms, bulky disease, radiotherapy, and risk stratification (Table 3).

**Table 3:** Comparison of baseline characteristics between the BV‐AVD + R group and the BV‐AVD group.

		BV‐AVD + R (*n* = 33)	BV‐AVD (*n* = 10)	*p*
Age (median, min–max)		11 (5–16)	19.5 (14–25)	0.003
Sex (%)	Male	22 (66.7)	5 (50.0)	0.339
	Female	11 (33.3)	5 (50.0)	
Histology (%)	Nodular sclerosis	12 (36.4)	6 (60.0)	0.362
	Mixed cellularity	12 (36.4)	2 (20.0)	
	Lymphocyte‐rich	1 (3.0)	1 (10.0)	
	Lymphocyte‐depleted	0 (0)	0 (0)	
	Unspecified	8 (24.2)	1 (10.0)	
Stage (%)	I	0 (0)	0 (0)	0.984
	II	9 (27.3)	3 (30.0)	
	III	14 (42.4)	4 (40.0)	
	IV	10 (30.3)	3 (30.0)	
B symptoms (%)	Yes	9 (27.3)	5 (50.0)	0.179
	No	24 (72.7)	5 (50.0)	
Bulky disease (%)	Yes	14 (42.4)	6 (60.0)	0.329
	No	19 (57.6)	4 (40.0)	
Radiotherapy (%)	Yes	9 (27.3)	2 (20.0)	0.644
	No	24 (72.7)	8 (80.0)	
Pediatric risk group (%)	Low risk	0 (0)	0 (0)	0.934
	Intermediate risk	7 (21.2)	2 (20.0)	
	High risk	26 (78.8)	8 (80.0)	
Adult risk group (%)	Favorable prognosis	0 (0)	0 (0)	0.979
	Unfavorable prognosis	9 (27.3)	3 (30.0)	
	IPS ≤ 3	21 (63.6)	6 (60.0)	
	IPS ≥ 4	3 (9.1)	1 (10.0)	

#### 3.2.2 Response and Survival Analysis

Overall ORR for BV‐containing regimens was 95.4%. BV‐AVD + R demonstrated 93.9% ORR (CRR 87.9%) versus 100% ORR (CRR 80.0%) for BV‐AVD alone, showing a significant ORR difference (*p* = 0.047). At a median follow‐up of 14 months (range: 3–29 months), the 2‐year PFS was 94.1% in the BV‐AVD + R subgroup and 88.2% in the BV‐AVD subgroup, with no significant difference observed (Figure 1B). The median follow‐up was 14 months (range: 3–26 months) for the BV‐AVD + R subgroup and 12.5 months (range: 6–29 months) for the BV‐AVD subgroup.

#### 3.2.3 Safety

Grade 3–4 adverse events were significantly higher with BV‐AVD + R (84.8% vs. 40.0%, *p* = 0.004), including hematological toxicity (78.8% vs. 20.0%, *p* = 0.023). Liver impairment occurred in 18.2% (all Grade ≥ 3) of BV‐AVD + R patients versus 60.0% (no Grade ≥ 3) with BV‐AVD (*p* = 0.004). Gastrointestinal symptoms and infection rates showed non‐significant differences (Table 4).

**Table 4:** Comparison of adverse events between BV‐AVD + R and the BV‐AVD regime.

	BV‐AVD + R (*n* = 33)		BV‐AVD (*n* = 10)		*p*	
	Incidence	Grade 3–4 incidence	Incidence	Grade 3–4 incidence	Incidence	Grade 3–4 incidence
Adverse events (%)	33 (100)	28 (84.8)	10 (100)	4 (40)	—	0.004
Hematological toxicity (%)	33 (100)	26 (78.8)	6 (60)	2 (20)	0.001	0.023
Liver impairment (%)	6 (18.2)	6 (18.2)	6 (60)	0 (0)	0.004	0.034
GI symptoms (%)	2 (6.1)	0 (0)	3 (30)	0 (0)	0.361	—
Infection (%)	2 (6.1)	1 (3)	3 (30)	2 (20)	0.414	0.513

Pre‐chemotherapy IgG levels were comparable between groups: BV‐AVD + R (14.2 g/L, range: 5.45–29.2) versus BV‐AVD (14.97 g/L, range: 10.88–22.62; *p* = 0.545). Post‐chemotherapy, BV‐AVD + R showed marked IgG reduction (4.95 g/L, range: 1.27–8.51) while BV‐AVD maintained levels (14.82 g/L, range: 11.15–16.35), with a significant intergroup difference (*p* < 0.001), suggesting that the inclusion of anti‐CD20 monoclonal antibody significantly impacted immune function, leading to a marked decline in IgG levels.

### 3.3 R/R CAYA HL Patients Receiving ICIs‐Containing Regimens

#### 3.3.1 Baseline Characteristics

Nineteen patients with R/R cHL treated with ICIs were enrolled: 9 from the Cancer Hospital of the Chinese Academy of Medical Sciences and 10 from Beijing Children's Hospital. The cohort comprised of 14 males and 5 females (median age: 15 years), predominantly presenting with advanced‐stage disease. All patients were stratified as high‐risk according to prognostic scoring systems. Nodular sclerosis was the most common histologic subtype, followed by mixed cellularity. Approximately half exhibited bulky disease (see Table 5).

**Table 5:** The baseline characteristics of patients receiving ICIs therapy.

		ICIs therapy (*n* = 19)
Age (median, min–max)		15 (3–24)
Sex (%)	Male	14 (73.7)
	Female	5 (26.3)
Histology (%)	Nodular sclerosis	10 (52.6)
	Mixed cellularity	5 (26.3)
	Lymphocyte‐rich	0 (0)
	Lymphocyte‐depleted	0 (0)
	Unspecified	4 (21.1)
Stage (%)	I	0 (0)
	II	5 (26.3)
	III	4 (21.1)
	IV	10 (52.6)
B symptoms (%)	Yes	11 (57.9)
	No	8 (42.1)
Bulky disease (%)	Yes	10 (52.6)
	No	9 (47.4)
Combination regimen	ICIs maintenance	8 (42.1)
	ICIs + BV‐AVD	1 (5.3)
	ICIs + IGEV‐BV	1 (5.3)
	ICIs + ABVD/AVD	3 (15.8)
	ICIs + CHOE	1 (5.3)
	ICIs + ICE	1 (5.3)
	ICIs + GP	1 (5.3)
	ICIs + GD	1 (5.3)
	ICIs + NP	1 (5.3)
	ICIs + decitabine + bendamustine	1 (5.3)

#### 3.3.2 Treatment Response and Survival Analysis

Among 19 evaluable patients, post‐treatment assessments revealed 11 CRs, 7 PRs, and 1 case of stable disease. With a median follow‐up of 36 months (range: 6–143 months), the 5‐year PFS rate was 47.4%. The 5‐year PFS was 63.6% for CR patients versus 28.6% for PR patients.

#### 3.3.3 Safety

Treatment tolerance was generally favorable. No infusion reactions (e.g. fever, chills, and rash) occurred during ICIs administration. No treatment delays, dose reductions, or discontinuations resulted from organ toxicity or severe adverse events. Two patients developed asymptomatic erythematous maculopapular eruptions on the neck post‐infusion, diagnosed by dermatology consultation as camrelizumab‐induced reactive cutaneous capillary endothelial proliferation. These resolved spontaneously without intervention after treatment cessation. Two patients experienced Grade ≥ 3 gastrointestinal symptoms. No Grade ≥ 3 adverse events were observed in thyroid, hepatic, renal, or cardiac function monitoring.

### 3.4 Comparison of Prognostic Scoring Systems

**Figure 2:**
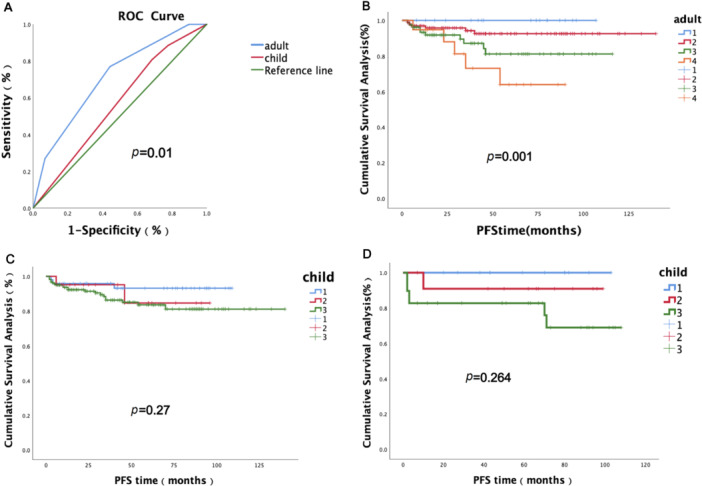
Selection of prognostic risk stratification models for HL patients under 24 years of age. (A) Comparative analysis of adult and pediatric prognostic scoring systems for Hodgkin's lymphoma patients aged 6–24 years; (B) progression‐free survival (PFS) curve analysis stratified by risk in patients aged 6–24 years with Hodgkin's lymphoma using the adult prognostic scoring system; (C) PFS curve analysis stratified by risk using the pediatric prognostic scoring system for Hodgkin's lymphoma patients aged 6–24 years; (D) PFS curve analysis stratified by risk using the pediatric prognostic scoring system for children with Hodgkin's lymphoma aged ≤ 6 years.

To more intuitively assess the accuracy of different scoring systems in adolescent and young adult (AYA) patients, we conducted an evaluation of each patient using both the adult scoring system and the pediatric Hodgkin's lymphoma scoring system. ROC curve analysis was employed to compare the predictive performance of these two systems for the outcomes of AYA Hodgkin's lymphoma. The areas under the ROC curve (AUC) for the adult and pediatric scoring systems were 0.696 and 0.573, respectively, with a statistically significant difference (*p* = 0.01). These findings indicate that the adult prognostic scoring system is more effective in predicting the prognosis of patients aged 6–24 years with Hodgkin's lymphoma. In the adult scoring system, the survival curve demonstrates that patients with favorable early prognostic scores exhibit the best outcomes, with a median PFS not reached and a 5‐year PFS rate of approximately 98.0%. Conversely, patients with an IPS score of ≥ 3 points have the worst prognosis, with a median PFS of approximately 69.8%. For children aged ≤ 6 years, due to the substantial differences between their serological indicators and those of adults, separate analyses were performed. The results indicate no significant difference when applying adult risk stratification (*p* = 0.27), while child‐specific risk stratification revealed trends in survival curves, though these differences were not statistically significant (*p* = 0.264) (Figure 2).

## 4 Discussion

HL constitutes a rare malignant neoplasm primarily involving lymph nodes and the lymphatic system, accounting for approximately 15%–25% of all lymphomas [12]. The disease exhibits a bimodal age distribution, with the initial peak occurring in CAYA [13]. To date, no universally accepted consensus or standardized therapeutic protocol exists for this patient population [3, 14, 15].

The optimal treatment approach for CAYA HL remains contentious. Given limited evidence regarding diagnostic timelines, clinical trial enrollment rates, treatment adherence, and long‐term chemotherapy toxicities, current therapeutic recommendations predominantly reflect institutional preferences rather than prospective trial data [14]. Consequently, determining the most appropriate therapeutic strategy remains challenging. This retrospective analysis comparatively evaluates the efficacy and safety profiles of diverse regimens in CAYA cHL patients, aiming to establish a more scientifically rigorous foundation for clinical decision‐making [13].

BV represents an antibody–drug conjugate that selectively targets CD30‐expressing tumor cells. Following internalization via endocytosis, lysosomal proteases cleave the linker, releasing monomethyl auristatin E (MMAE) [16, 17]. MMAE inhibits tubulin polymerization, disrupting mitosis and inducing tumor cell apoptosis. When combined with conventional chemotherapy (e.g., BV‐AVD evaluated in the ECHELON‐1 trial for advanced cHL) [18, 19, 20], this regimen demonstrated superior PFS and overall survival versus ABVD. However, novel agent exploration in CAYA remains limited. In our cohort, 43 cHL patients received frontline BV‐containing regimens. Compared to conventional chemotherapy, treatment efficacy showed no significant difference [5, 21]. Although PFS curves demonstrated divergence, 2‐year rates lacked statistical significance, potentially attributable to shorter BV group follow‐up. Regarding safety, Grade 3–4 hematological toxicity incidence was comparable, but conventional therapy exhibited significantly higher Grade 4 events. Notably, unlike clinical trial observations, our real‐world data revealed no increased peripheral neuropathy incidence with targeted therapy [5, 22, 23].

Some pediatric oncologists believe that although Reed‐Sternberg cells themselves rarely express CD20, the tumor microenvironment contains a significant number of CD20‐positive regulatory B cells, which may have immunosuppressive effects. Rituximab, by depleting these cells, could potentially improve antitumor immune responses. The Phase II trial by Hochberg et al. suggests that incorporating anti‐CD20 monoclonal antibodies into BV‐AVD may enhance therapeutic efficacy [24]. Their cohort (*n* = 30, median age 15 years) achieved 100% CR, with 60% attaining early metabolic response and 100% 5‐year event‐free/overall survival. However, this viewpoint has not gained widespread acceptance in adult treatment centers. Based on real‐world data from two centers, we sought to provide preliminary observational evidence regarding whether the addition of rituximab to BV‑AVD offers additional benefit for specific CAYA patients. Although there was a difference in age distribution between the two groups, this disparity did not occur randomly but rather stemmed from different treatment concepts and perspectives. The results showed that BV‑AVD + R compared with BV‑AVD alone yielded a higher ORR in the BV‑AVD group, but no significant survival advantage was observed. However, the follow‐up duration for the BV‐AVD group was relatively short. This difference in follow‐up time may lead to an underestimation of the long‐term recurrence risk and delayed toxicities associated with the BV‐AVD regimen, thereby affecting the assessment of the true PFS difference between the two groups. Longer follow‐up is needed to confirm the long‐term efficacy and safety. Safety evaluation indicated that the BV‑AVD + R regimen significantly increased Grade 3–4 hematologic toxicity and was associated with a higher incidence of severe liver injury. The addition of the anti‑CD20 antibody markedly reduced post‑treatment immunoglobulin levels, thereby increasing the risk of infection without conferring therapeutic benefit. Nevertheless, further validation through prospective studies is still required. This is preliminary efficacy data observed in this real‐world cohort. The observed trend toward reduced hematological toxicity with BV‐based regimens may be due to differences in baseline performance status, economic factors, and comorbidities among the patient population able to receive the more expensive novel therapy, potentially leading to a lower incidence of hematological toxicity.

Studies including CHECKMATE‐205 and KEYNOTE‐087 demonstrate that ICIs achieve an ORR of 70% in adults with R/R cHL [25, 26, 27]. To establish pediatric ICI dosing, a study enrolled 154 HL patients aged 6 months to 17 years, including 15 with R/R HL treated with pembrolizumab [19]. The ORR was 80%, with safety analysis revealing Grade 3–5 adverse events in 69 patients (45%) and serious adverse events (SAEs) in 14 [25, 28, 29], most commonly fever, hypertension, and pleural effusion [4, 26]. While clinical trials indicate single‐agent ICIs confer effective antitumor activity and acceptable safety, their real‐world application in CAYA populations—particularly pediatric patients—remains constrained by multiple factors [30]. This study included 19 R/R HL patients aged 3–24 years receiving ICI‐containing regimens. Fifteen presented with advanced‐stage disease, and 12 had bulky lesions. ICIs were generally well‐tolerated, with no observed significant organ dysfunction (thyroid, hepatic, renal, or adrenal/pituitary). As a retrospective analysis of ICIs‐treated CAYA HL cases from two medical centers, this study has a limited sample size and heterogeneous combination chemotherapy regimens. Further research is warranted to establish optimal therapeutic strategies for this population.

For patients with cHL, the selection of an appropriate prognostic scoring system remains a subject of debate. The prognostic model developed by the Children's Oncology Group (COG) included only disease stage, presence of a large mass, and B symptoms. Additionally, the team led by Geoerger et al. identified that for cHL patients, factors such as Stage IV disease, the presence of a large mediastinal mass, albumin levels below 35 g/L, and fever were independent predictors of event‐free survival [29]. This study aimed to compare the prognostic assessment results of two distinct scoring systems in patients with CAYA cHL. The findings indicated that the adult IPS scoring system exhibited relatively higher accuracy in predicting PFS among patients with CAYA cHL [20]. In contrast to the pediatric risk stratification system, the adult IPS scoring system demonstrated more pronounced statistical differences in differentiating PFS rates across various risk levels and was capable of predicting PFS status with greater precision [31]. These results suggest that the adult IPS scoring system may be more suitable and possess higher clinical applicability for prognostic assessments in patients with CAYA cHL. Regarding the enhancement of the prognostic scoring system, it is necessary to integrate additional biomarkers and clinical factors to develop more accurate prognostic prediction models.

## 5 Conclusion

In conclusion, this study underscores the promising role of BV‐AVD‐based regimens in the treatment of CAYA Hodgkin lymphoma. Furthermore, the superior performance of adult prognostic scores in the 6–24‐year cohort highlights the need for age‐appropriate risk stratification. Lastly, the high efficacy and favorable safety of ICIs in the relapsed/refractory setting offer a valuable treatment option for this challenging population. These findings collectively support a risk‐adapted, efficacy‐driven approach to optimize outcomes in CAYA Hodgkin lymphoma.

## 6 Limitation

The optimal treatment strategy for CAYA HL remains controversial. The key innovation and primary contribution of this study lie in its exploration of the evidence gap regarding the real‐world use of novel treatment regimens within China's specific population, thereby providing a preliminary localized evidence base for the application of these regimens in Chinese clinical practice. Furthermore, for this population lacking unified standards, this study preliminarily investigated the potential applicability and limitations of existing prognostic risk scoring systems. We confirmed that the AUC values did not reach a high level, indicating that both risk scoring systems have limited ability to differentiate prognosis in this CAYA cohort. Clinicians should formulate individualized therapeutic strategies based on patient‐specific factors to optimize efficacy while enhancing prognosis and quality of life. Future initiatives should prioritize clinical research advancement to identify more precise and effective therapeutic approaches.

## Author Contributions

**Mengqing Xie:** investigation (lead), methodology (lead), software (lead), data curation (lead), writing – original draft (lead). **Meng Yuan:** investigation (lead), methodology (lead), software (lead), formal analysis (lead), visualization (lead), data curation (lead), writing – original draft (lead). **Mengwei Ren:** writing – review and editing (lead), data curation (lead), software (lead), investigation (lead) conceptualization (lead). **Jing Tian:** visualization (supporting), writing – review and editing (supporting), software (supporting), data curation (supporting). **Shengyu Zhou:** resources (supporting), project administration (supporting), visualization (supporting). **Xiaohui He:** resources (supporting), project administration (supporting), visualization (supporting). **Yan Qin:** project administration (supporting), resources (supporting), validation (supporting). **Peng Liu:** validation (supporting), project administration (supporting), resources (supporting). **Jianliang Yang:** validation (supporting), project administration (supporting), resources (supporting). **Mengyuan Han:** methodology (supporting), software (supporting), data curation (supporting), investigation (supporting). **Fei Ma:** resources (supporting), project administration (supporting), funding acquisition (supporting). **Sheng Yang:** resources (supporting), project administration (supporting), writing – review and editing (supporting), funding acquisition (supporting). **Sidan Li:** writing – review and editing (lead), project administration (lead), resources (lead), supervision (lead), funding acquisition (lead), conceptualization (lead), methodology (lead), validation (lead).

## Ethics Statement

This study is a retrospective investigation that solely involves the collection and analysis of existing anonymized medical records and data. It does not entail any interventional procedures and poses no additional risks to the rights, welfare, or health of the subjects. The design and implementation of this study protocol strictly adhere to the ethical principles for medical research involving human subjects as set forth in the Declaration of Helsinki. This study was approved by Ethics Committee of National Cancer Center/Cancer Hospital, Chinese Academy of Medical Sciences and Peking Union Medical College (Approval number 26/030‐0353).

## Consent

From all patients included in this study, written informed consent was obtained, allowing us to analyze the data from their electronical health records and to publish data anonymized. All analyses were performed retrospectively. No experiments on humans or use of human tissue samples have been performed.

## Conflicts of Interest

Professor Fei Ma is a member of the *Cancer Innovation* Editorial Board. To minimize bias, he was excluded from all editorial decision‐making related to the acceptance of this article for publication.

## Supporting information

Appendix 1

## Data Availability

The data that support the findings of this study are available from the corresponding author upon reasonable request.
