# Supplementary figures and images for: Treatment Outcomes With Novel Targeted and Immunotherapeutic Regimens in CAYA Hodgkin Lymphoma: A Retrospective Study
Source: Cancer Innov. 2026 Mar 16;5(2):e70050. doi: 10.1002/cai2.70050 (PMC13070277; doi:10.1002/cai2.70050)

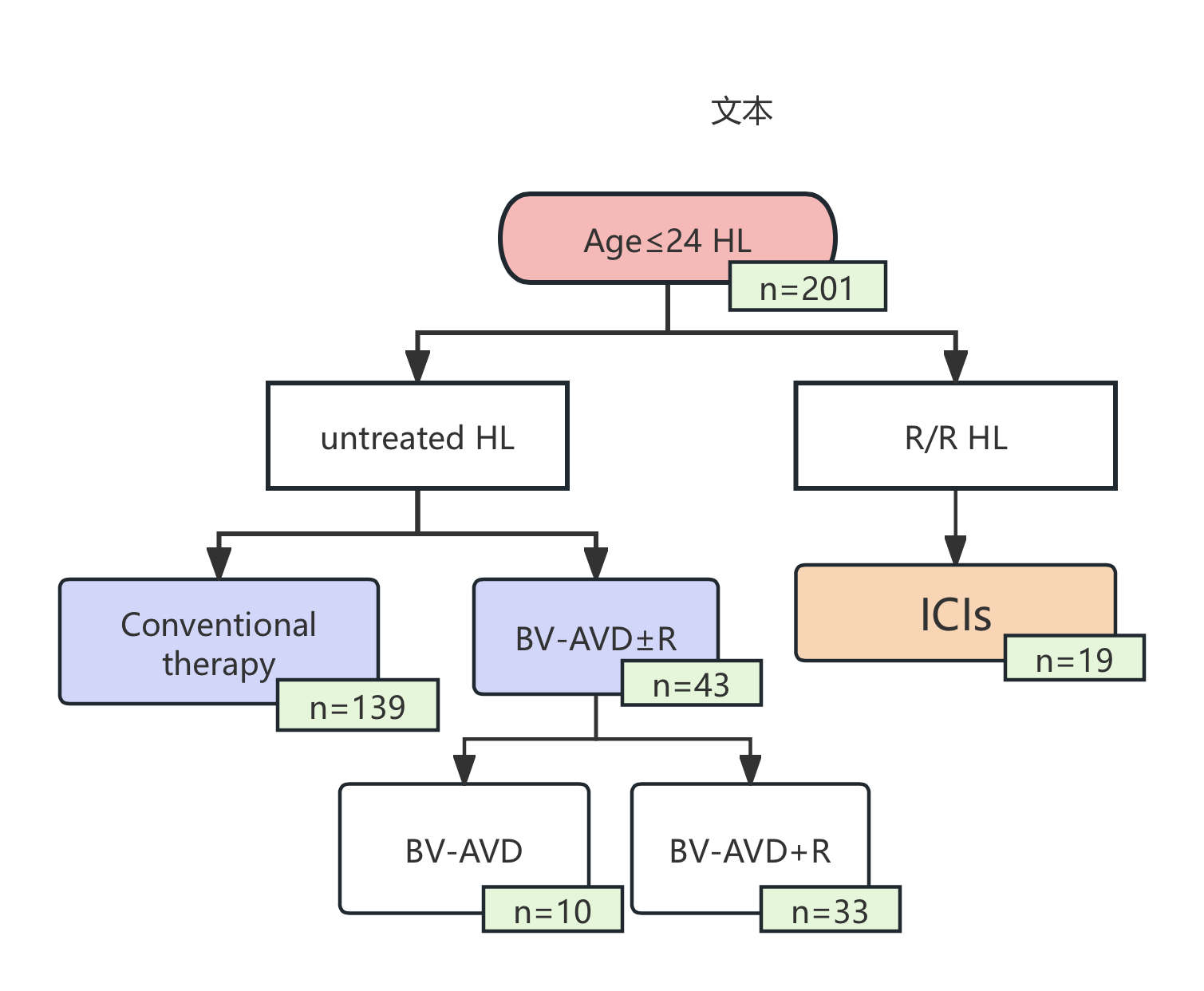

Supplement: Supplementary file 1 — Appendix 1 [file CAI2-5-e70050-s001.docx]
